# Thrombin-Derived C-Terminal Peptide Reduces *Candida*-Induced Inflammation and Infection *In Vitro* and *In Vivo*

**DOI:** 10.1128/AAC.01032-21

**Published:** 2021-10-18

**Authors:** Anna Dahlman, Manoj Puthia, Jitka Petrlova, Artur Schmidtchen, Ganna Petruk

**Affiliations:** a Xinnate AB, Medicon Village, Lund, Sweden; b Division of Dermatology and Venereology, Department of Clinical Sciences, Faculty of Medicine, Lund University, Lund, Sweden; c Copenhagen Wound Healing Center, Bispebjerg Hospital, Department of Biomedical Sciences, University of Copenhagen, Copenhagen, Denmark; d Dermatology, Skåne University Hospital, Lund, Sweden

**Keywords:** thrombin, *Candida*, antifungal, zymosan, antimicrobial peptide, antifungal agent

## Abstract

Infections due to the opportunistic fungus Candida have been on the rise in the last decades, especially in immunocompromised individuals and hospital settings. Unfortunately, the treatments available today are limited. Thrombin-derived C-terminal peptide (TCP-25) is an antimicrobial peptide (AMP) with antibacterial and immunomodulatory effects. In this work, we, for the first time, demonstrate the ability of TCP-25 ability to counteract Candida *in vitro* and *in vivo*. Using a combination of viable count assay (VCA), radial diffusion assay (RDA), and fluorescence and transmission electron microscopy analyses, TCP-25 was found to exert a direct fungicidal activity. An inhibitory activity of TCP-25 on NF-κB activation induced by both zymosan alone and heat-killed C. albicans was demonstrated *in vitro* using THP-1 cells, and *in vivo* using NF-κB reporter mice. Moreover, the immunomodulatory property of TCP-25 was further substantiated *in vitro* by analyzing cytokine responses in human blood stimulated with zymosan, and *in vivo* employing a zymosan-induced peritonitis model in C57BL/6 mice. The therapeutic potential of TCP-25 was demonstrated in mice infected with luminescent C. albicans. Finally, the binding between TCP-25 and zymosan was investigated using circular dichroism spectroscopy and intrinsic fluorescence analysis. Taken together, our results show that TCP-25 has a dual function by inhibiting *Candida* as well as the associated zymosan-induced inflammation. The latter function is accompanied by a change in secondary structure upon binding to zymosan. TCP-25, therefore, shows promise as a novel drug candidate against *Candida* infections.

## INTRODUCTION

The increase in antimicrobial resistance poses a serious problem to public health. Although bacteria are responsible for the majority of infections, fungal manifestations are also occurring and are difficult to treat. It is estimated that over a billion people are affected, and 1.5 million deaths can be associated with fungal infections worldwide (1, 2).

Several *Candida* species are responsible for the majority of the fungal infections today, with Candida albicans constituting more than half of these (3–6). *Candida* is usually a commensal, participating in the ordinary flora in the gastrointestinal, respiratory, and genital tracts. *Candida* may, however, also act as a pathogen in immunologically weak and immunocompromised people. Indeed, this microorganism is often described as “opportunistic,” with a shift from commensal to pathogen (7, 8). The treatments available today for fungal infections are limited and provide low diversity in their mechanism of action because fungi and mammalian cells are very similar at the biochemical and biological levels (9). Due to the limitations of these treatments, resistance development among *Candida* species is a major problem (10). Hence, the need for new treatments is apparent.

Antimicrobial peptides (AMPs) have recently gained much attention as new alternatives to classical antibiotics (11–14). AMPs are a widespread class of molecules that are derived from the host’s natural defense system and constitute an important branch of the innate immune system, which is the first line of defense toward pathogens (15). Two common qualities of AMPs apply among all phyla, namely, they are usually cationic and amphipathic (16). These two features enable the peptides to have a broad range of activities, as they allow them to act on the entire cellular membrane (10, 16) or through other complex mechanisms (17). This, in turn, results in lower development of antimicrobial resistance, due to a relatively low risk of evolution of changes in the membrane charge of the pathogen and/or the release of extracellular proteases that can cleave the AMPs (17–21). Apart from antimicrobial properties, the AMPs can also have immunomodulatory and wound healing properties (22). Most of the AMPs known today are specific for bacteria but could potentially be effective against fungal infections as well (10).

We have previously investigated AMPs derived from thrombin with bactericidal and anti-inflammatory properties (10, 23–28). Thrombin is a large protein of about 36 kDa that plays an important role in the coagulation cascade necessary for wound healing. During the clotting process, thrombin undergoes a series of cleavage processes and forms smaller active fragments (29). Further proteolysis leads to the formation of fragments of about 11 kDa, which mediate the aggregation of lipopolysaccharide (LPS) and bacteria, facilitating endotoxin clearance and microbial killing (25, 30). An even shorter version of around 2 kDa can be produced, which is characterized by strong antibacterial and anti-inflammatory activity (23, 24, 26–28). The 25-amino-acid thrombin-derived C-terminal peptide (TCP-25), GKYGFYTHVFRLKKWIQKVIDQFGE, has been extensively characterized (23, 24, 26–28).

TCP-25 assumes an α-helical structure upon binding to LPS and lipid membranes, which leads to destabilization and disruption of the bacterial membrane (24). Previously, it was demonstrated that TCP-25 inhibits LPS-induced NF-κB activation by binding to LPS. In addition, TCP-25 binds competitively to the hydrophobic pocket of CD14, preventing TLR4 dimerization (28). A hydrogel with TCP-25 as an active ingredient exerted antibacterial and immunomodulatory actions, suggesting that this “dual action” effect could potentially be used in clinical settings (27). Whether the anti-infective capacity of TCP-25 could be further extended to fungi, however, remained to be explored.

Here, the antifungal activity of TCP-25 was explored using different species of *Candida*, i.e., C. albicans, Candida parapsilosis, Candida krusei, Candida glabrata, and Candida lipolytica (also known as Yarrowia lipolytica). Using fluorescence microscopy and transmission electron microscopy (TEM), we show that TCP-25 kills yeasts through disruption of their membrane. Moreover, TCP-25 abrogates NF-κB activation in response to heat-killed yeast or yeast-derived zymosan. The peptide also reduces cytokine levels in human blood stimulated by zymosan as well as in mice using an experimental model of zymosan-induced peritonitis. In an infection model in mice, TCP-25 treats *Candida* infection. Finally, using different biophysical techniques, we characterize the binding between TCP-25 and zymosan.

## RESULTS

### Antifungal activity of TCP-25.

**FIG 1:**
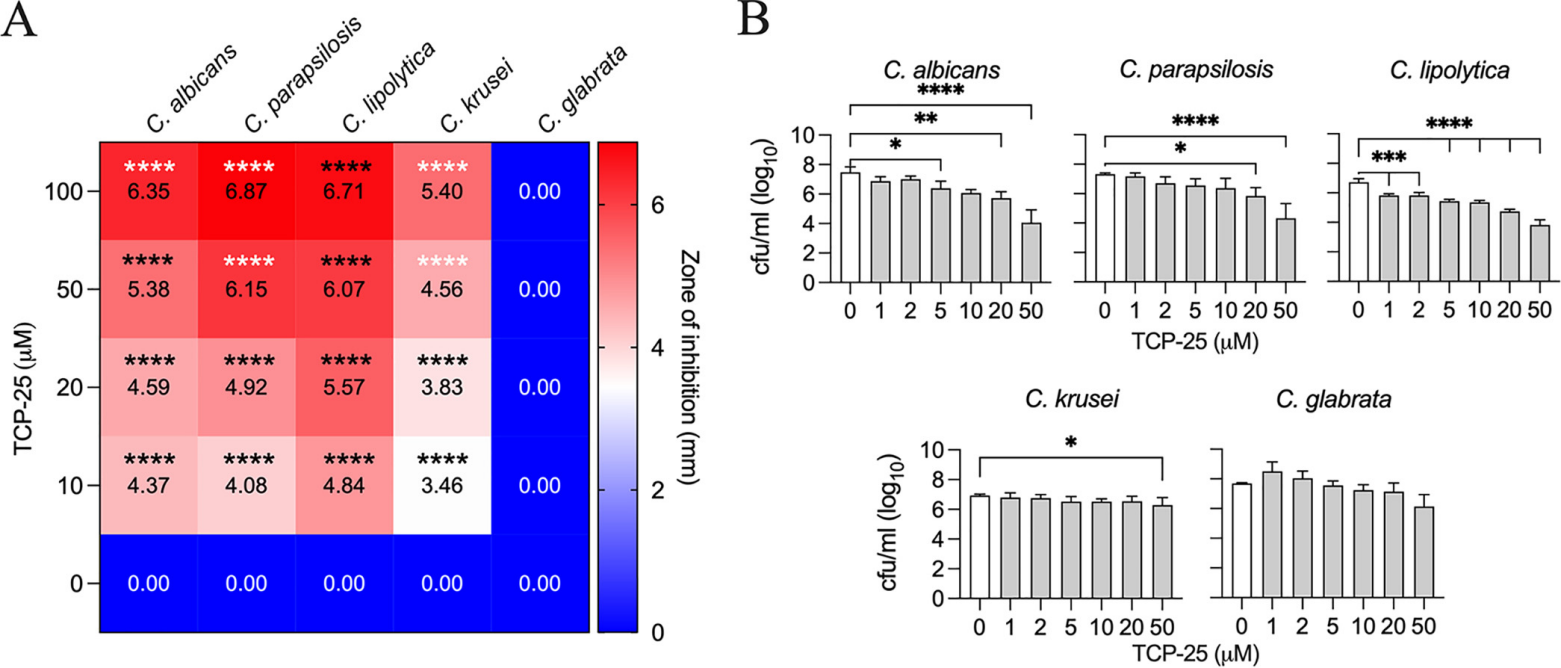
Antifungal activity of TCP-25. (A) Heatmap illustrating the inhibitory effect of TCP-25 as zones of clearance of C. albicans, C. parapsilosis, *C. lipolytica*, C. krusei, and C. glabrata. TCP-25 was tested at 0, 10, 20, 50, and 100 μM. Color and values in each box represent mean values in millimeters (*n* = 3). (B) Killing of different *Candida* species in solution by TCP-25 was investigated by VCA. Increasing concentrations of TCP-25 (0 to 50 μM) were incubated for 2 h with the same amount of yeast cells, then plated. The day after, CFU/ml were counted. Data are presented as mean ± standard deviation (SD) (*n* = 3). Significance was determined for each treatment with respect to the untreated sample by using one-way analysis of variance (ANOVA) followed by Dunnett’s multiple-comparison test using GraphPad Prism software. *, *P ≤ *0.05; **, *P ≤ *0.01; ***, *P ≤ *0.001; ****, *P ≤ *0.0001.

TCP-25 exerts antimicrobial activity against Gram-negative and Gram-positive bacteria in both *in vitro* and *in vivo* settings (27, 28, 31). Using a radial diffusion assay (RDA), we demonstrated that TCP-25 was also active against several *Candida* species. A dose-dependent increase in the zone of clearance was observed (Fig. 1A; see also Fig. S1 in the supplemental material for illustration). Interestingly, no effect was seen on C. glabrata. The activity of TCP-25 in solution was determined by viable count assay (VCA), where C. albicans, C. parapsilosis, and C. lipolytica all showed a significant and dose-dependent decrease in the CFU count, with increasing concentration of the peptide (Fig. 1B). Reduction of C. krusei was only observed at the highest concentration used (50 μM peptide), while no significant effect was seen on C. glabrata. Overall, the results from the two methods demonstrate an antifungal potential of the peptide on selected *Candida* species.

### Permeabilization and morphological alterations in the yeast membrane induced by TCP-25.

**FIG 2:**
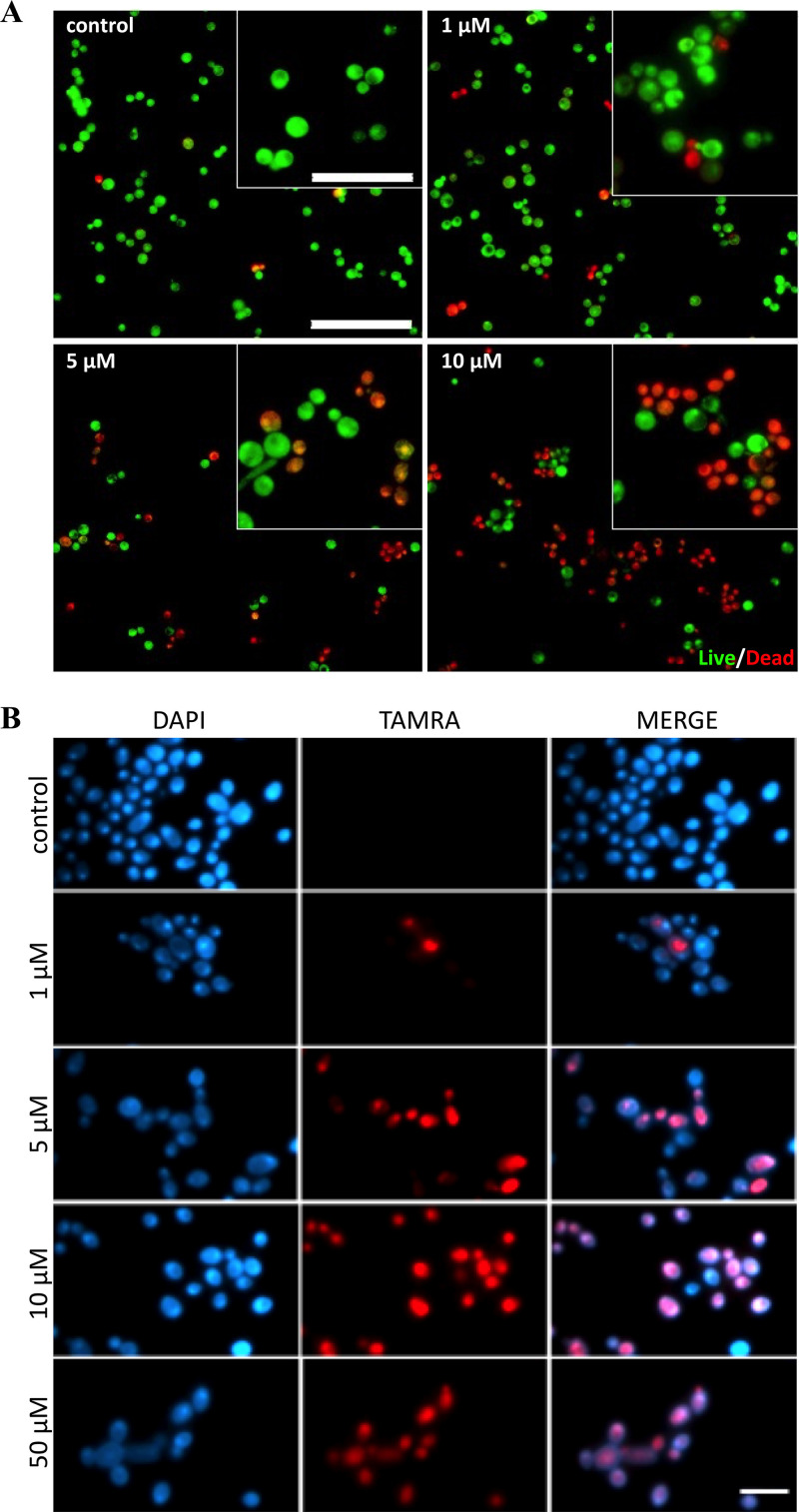
Fluorescence microscopy of Candida albicans cells (A) C. albicans was treated with increasing concentrations of TCP-25 (0 to 10 μM) for 2 h, and then a live/dead assay was performed. Representative pictures for each treatment from three independent experiments are shown (10 individual fields per experiment, *n* = 3). Dead yeast cells are visualized in red, and alive cells in green. Bar, 50 μm for all images and 20 μm for the inserts. (B) C. albicans was treated with increasing concentrations of tetramethylrhodamine (TAMRA)-labeled TCP-25 (0 to 50 μM) for 2 h. The images were taken after staining of yeast cells with 4′,6-diamidino-2-phenylindole (DAPI). Representative pictures for each treatment from three independent experiments are shown (at least 10 individual fields per experiment, *n* = 3). Yeast cells are visualized in blue (DAPI); labeled TCP-25 is visualized in red (TAMRA). Bar, 10 μm.

To understand if the effect of TCP-25 was mediated by membrane disruption, and consequently permeabilization to propidium iodide, a live/dead assay was performed. Based on the above-described results, two species of *Candida* were chosen, C. albicans and C. parapsilosis. Yeast cells were analyzed by fluorescence microscopy after incubation with buffer only or with increasing concentrations of TCP-25. The result for C. albicans is displayed in Fig. 2A, while data for C. parapsilosis are presented in Fig. S2A. In both cases, the relative number of yeast cells with red fluorescence increases with higher doses of the peptide, indicating loss of membrane integrity. Transmission electron microscopy (TEM) analysis of C. albicans cells subjected to TCP-25 treatment showed that the peptide caused profound morphological changes. Several blebs (black arrow) were clearly visible in the images, with protrusions (blue arrow) and leakage of electron-dense material (red arrow) (Fig. S2B), results compatible with those of previous reports on other membrane-active peptides (32, 33). In order to further investigate the peptide’s mode of action, we incubated C. albicans with increasing doses of tetramethylrhodamine (TAMRA)-labeled TCP-25, followed by staining with 4′,6-diamidino-2-phenylindole (DAPI) and analysis by fluorescence microscopy. Results showed that TCP-25 dose dependently bound to C. albicans (Fig. 2B), and at lower concentrations, we observed a staining in intracellular structures, whereas higher peptide concentrations yielded a diffuse staining (Fig. S2C). Taken together, the results show that TCP-25 can bind to and permeabilize *Candida* cells.

### TCP-25 inhibits zymosan-induced NF-κB activation in monocytes and reduces cytokines in human blood *in vitro* and in an *in vivo* model in mice.

**FIG 3:**
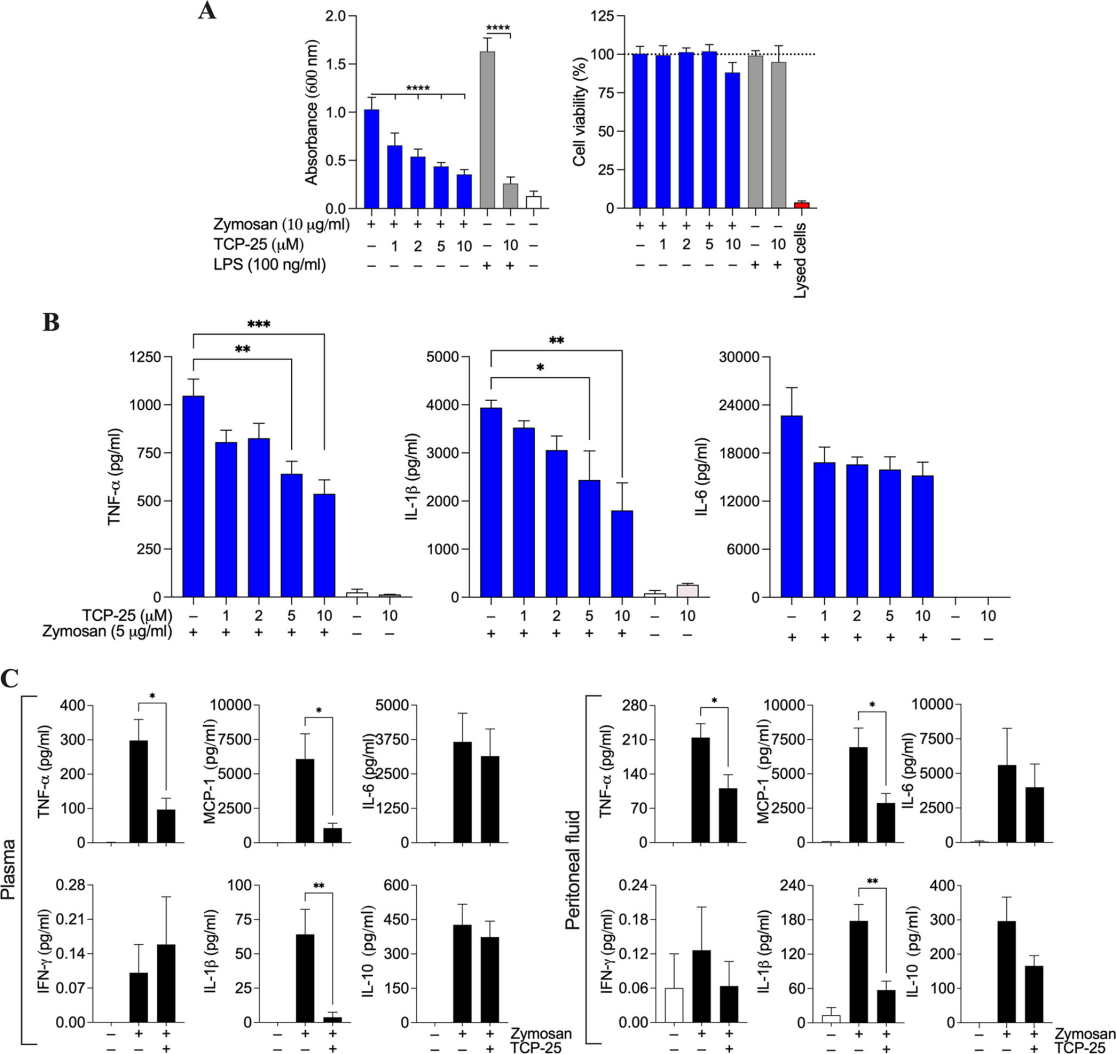
TCP-25 counteracts zymosan-induced inflammation *in vitro* and *in vivo*. (A) Monocytes were incubated with an increasing concentration of zymosan and then NF-κB activation (left) and cell viability (right) were measured. Lipopolysaccharide (LPS) (100 ng/ml) without and with TCP-25 (10 μM) was used for comparison. Results are presented as the mean of absorbance or percentage (with respect to untreated cells, 100%) ± SD of 4 independent experiments (*n* = 4). Significance is expressed in comparison to cells treated with only zymosan or LPS, and was obtained using an ordinary one-way ANOVA followed by Dunnett’s multiple-comparison test. ****, *P ≤ *0.0001. The dashed line indicates the vitality of untreated cells. (B) Whole blood was stimulated with zymosan (10 μg/ml) in the presence or absence of different doses of TCP-25 (0 to 10 μM). After 24 h of incubation, tumor necrosis factor alpha (TNF-α), interleukin 1 beta (IL-1β), and IL-6 release levels were analyzed. The results are presented as mean ± standard error of the mean (SEM) of 3 independent experiments, each done with blood from different donors (*n* = 3). Significance is expressed in comparison to blood treated only with zymosan and was obtained using an ordinary one-way ANOVA followed by Dunnett’s multiple-comparison test. *, *P ≤ *0.05; **, *P ≤ *0.01; ***, *P ≤ *0.001. (C) The anti-inflammatory activity of TCP-25 *in vivo* was studied by interperitoneally (i.p.) injecting 1 mg zymosan per mouse in C57BL/6 mice. Thirty minutes after zymosan injection, 0.5 mg TCP-25 in 10 mM NaOAc at pH 5 or buffer alone (control) was injected i.p. into the mice. After 4 h of zymosan stimulation, the animals were deeply anesthetized by isoflurane; then, blood and peritoneal fluid were collected and cytokine release was analyzed (*n* = 3 for control, *n* = 6 to 8 for treated animals). Significance is expressed in comparison to animals treated only with zymosan and was obtained using an ordinary one-way ANOVA followed by Dunnett’s multiple-comparison test. *, *P ≤ *0.05; **, *P ≤ *0.01.

It has been reported that zymosan binds to Toll-like receptor 2 (TLR-2) on the surface of immune cells and triggers the MAP-kinase cascade and subsequent NF-κB signaling and, thereby, promotes inflammation (34). Having shown the antifungal activity of TCP-25, we next investigated whether TCP-25 could suppress the proinflammatory activity of zymosan. For this, we used THP1-XBlue-CD14 cells that stably express CD14 and MD2, apart from Toll-like receptors. The monocytes were exposed to increasing concentrations of zymosan to find the right dose (Fig. S3). The cells were then treated with 10 μg/ml zymosan in the presence of different concentrations of the peptide. The result showed that TCP-25 dose dependently inhibited NF-κB activation induced by zymosan (Fig. 3, left). In fact, all concentrations of TCP-25 showed a significant zymosan-quenching effect. The cell viability assay showed no significant cytotoxic effect of TCP-25 on THP-1 cells (Fig. 3, right). Therefore, the reduction in the NF-κB activation at all peptide concentrations is attributable to the inhibitory effect of TCP-25 on zymosan.

The reduction of zymosan-mediated inflammation was further investigated in human blood, providing a relevant experimental system mimicking the physiological situation. Blood was stimulated with 5 μg/ml of zymosan in the presence or absence of TCP-25, followed by enzyme-limited immunosorbent assay (ELISA) to monitor inflammatory cytokines (Fig. 3B). A dose-dependent decrease in cytokine levels could be seen for tumor necrosis factor alpha (TNF-α), interleukin 1 beta (IL-1β), and IL-6.

To verify if zymosan-induced inflammation was counteracted by TCP-25 *in vivo* as well, we used a zymosan-induced peritonitis model in C57BL/6 mice. Animals were treated with 1 mg of zymosan per mouse (35), and 30 min later, TCP-25 was injected intraperitoneally (i.p.). Inflammation was evaluated by detecting changes in the cytokine levels in plasma and peritoneal fluid collected from the mice after 4 h. As shown in Fig. 3C, TNF-α, MPC-1, and IL-1β were reduced upon treatment with TCP-25, both in plasma and in peritoneal fluid. No significant reduction was seen for IL-6, which might be explained by the fact that it is a cytokine secreted in the late phase. IL-10, on the other hand, was increased in both mice treated with zymosan alone and those treated with zymosan with TCP-25. This is not surprising, since it was reported previously that zymosan stimulates abundant secretion of IL-10 (36), and since it is an anti-inflammatory cytokine, high levels can be expected in treatment with TCP-25 (37). Altogether, the results for plasma and intraperitoneal fluid from this experiment were compatible with the data from THP-1 cells and human blood, demonstrating that TCP-25 can reduce zymosan-induced inflammation.

### TCP-25 inhibits heat-killed C. albicans-induced NF-κB activation in monocytes and in an *in vivo* model in mice.

To further substantiate the immunomodulatory properties of TCP-25 against *Candida*, we decided to evaluate its activity by inducing NF-κB production using C. albicans cells *in vitro* and *in vivo*. In order to simulate a situation in which killed *Candida* triggers proinflammatory responses, and to avoid *Candida* growth and invasion as an experimental confounder, we decided to use a reductionist approach, employing heat-killed C. albicans (HKCA). Different methods of obtaining HKCA are reported in the literature, and we used treatment at 80°C and 100°C for different lengths of time (38, 39). Both methods yielded no yeast growth after plating on Sabouraud dextrose agar (SDA) plates (Fig. S4A). We next evaluated NF-κB activation by these two preparations of HKCA on THP-1 cells, and used zymosan as the positive control. The results showed that HKCA obtained with both methods induce inflammation, but that generated at 80°C yielded a stronger response in the THP-I cells (Fig. S4B). We therefore decided to stimulate THP-1 cells with HKCA generated at 80°C in the absence or the presence of increasing doses of TCP-25. After 24 h of incubation, NF-κB activation and cell viability were evaluated (Fig. S4C). A significant reduction in NF-κB activation was already observed at low concentrations of TCP-25. The *in vitro* results were confirmed by using an experimental mouse model of subcutaneous inflammation (Fig. S5). Indeed, a significant reduction in HKCA-stimulated NF-κB activation was observed in the NF-κB reporter mice treated with TCP-25 at both time points.

### Effects of zymosan on the conformation of TCP-25.

**FIG 4:**
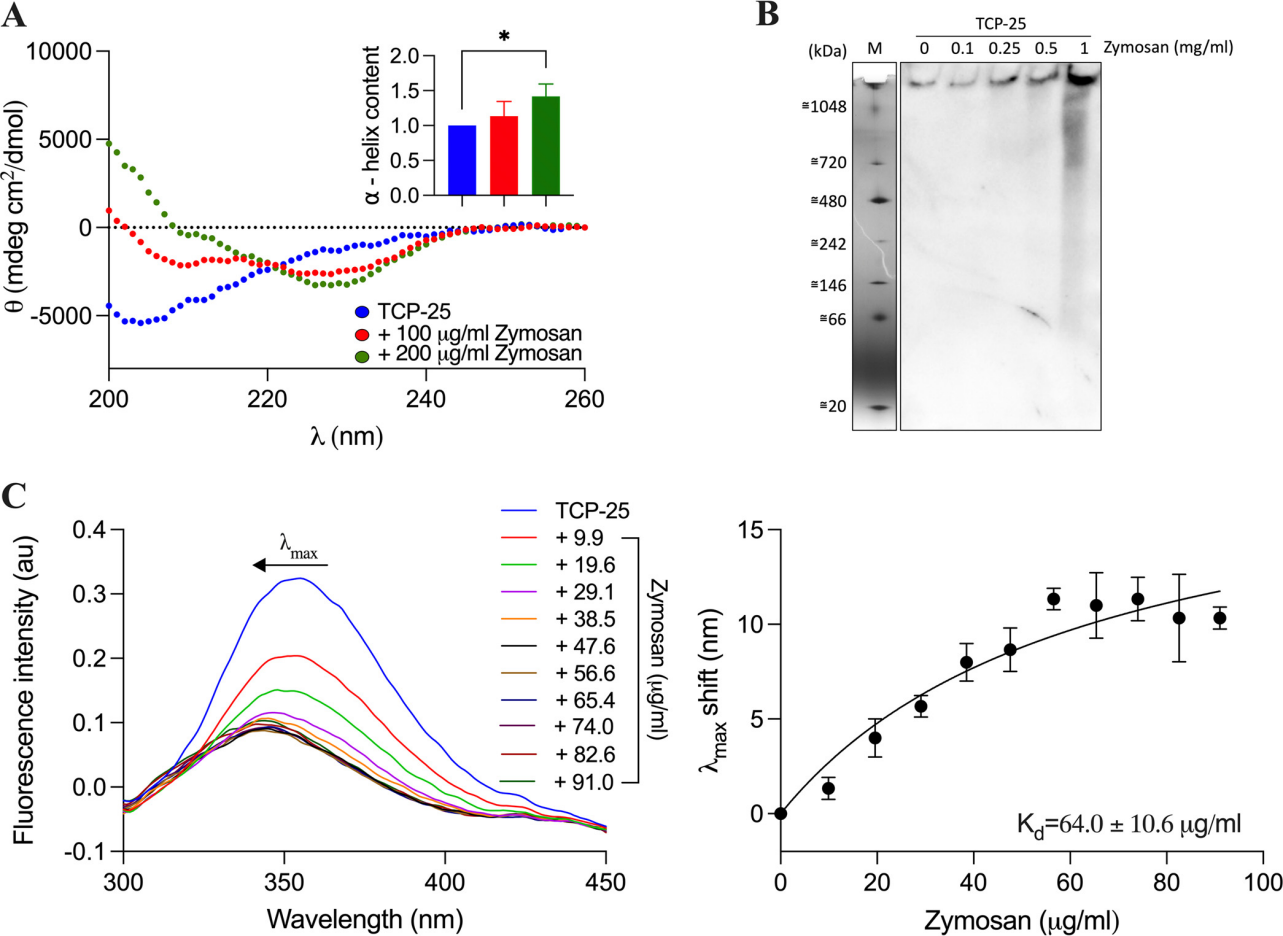
Structural changes of TCP-25 upon binding to zymosan. (A) The secondary structure of TCP-25 (10 μM) alone and in the presence of 100 and 200 μg/ml zymosan was studied by circular dichroism (CD). Measurements were performed immediately after the addition of zymosan. Representative spectra of 3 different experiments are reported (*n* = 3). The α-helical content at 222 nm was calculated from the spectra obtained for each condition. Results are expressed as mean ± SD. Significance was established by an ordinary one-way ANOVA followed by Dunnett’s multiple-comparison test using GraphPad Prism software. *, *P ≤ *0.05. (B) TCP-25 (2 μg) was incubated with increasing doses of zymosan (0 to 1.0 mg/ml) for 30 min at 37°C. Then, the mixture was analyzed by clear native (CN)-PAGE followed by Western blot using antibodies against the C-terminal region of TCP-25. One representative image from 3 independent experiments is shown (*n* = 3). (C) The binding affinity of zymosan to TCP-25 was studied by measuring the intrinsic fluorescence of the peptide. TCP-25 (10 μM) was titrated with increasing doses of zymosan (9.9 to 91.0 μg/ml); then, the sample was exposed to 280 nm excitation, and the emission spectra between 300 and 450 nm were acquired using a spectrofluorometer. Representative spectra of 3 independent experiments are reported (*n* = 3). The saturation curve was obtained reporting a shift of emission maxima (λ_max_) in the function of different concentrations of zymosan. The results are expressed as mean ± SD of 3 independent experiments (*n* = 3).

Given that TCP-25 blocks zymosan’s proinflammatory activity *in vitro* and *in vivo*, we hypothesized that the peptide binds to zymosan. To study this, the secondary structure of TCP-25 alone or in the presence of 100 or 200 μg/ml zymosan was investigated using circular dichroism (CD) spectroscopy. The spectrum of TCP-25 alone showed a signal compatible with an unstructured conformation (Fig. 4A, blue line). When 100 μg/ml of zymosan was added to the peptide, a clear change in the secondary structure was observed (Fig. 4A, red line). This change was even more pronounced after the addition of 200 μg/ml zymosan (Fig. 4A, green line). The quantification of the change in the secondary structure was established by calculating the α-helical content at 222 nm as reported by Greenfield and Fasman (Fig. 4A, histograms) (40). To further corroborate these structural findings, we performed the analysis of secondary structure of TCP-25 in the presence of another glucan, i.e., Laminaria digitata laminarin, which yielded similar results to those seen for zymosan (Fig. S6). It should also be noted here that we have previously reported that TCP-25 assumes a helical shape when bound to Escherichia coli LPS (24, 41). The binding of TCP-25 to zymosan was further confirmed by native gel electrophoresis followed by Western blot (Fig. 4B). We incubated the peptide with increasing doses of zymosan and then evaluated its migration on the gel. Under the conditions used, TCP-25 does not enter the gel due to its high positive charge. However, upon the addition of zymosan, a dose-dependent shift in migration of TCP-25 was observed, indicative of binding.

Considering that TCP-25 contains one tryptophan and two tyrosine residues, respectively, we exploited its intrinsic fluorescence to study the binding affinity to zymosan. The result is displayed in Fig. 4C. The fluorescence emission maximum of unbound TCP-25 was found to be around 355 nm (Fig. 4C, blue line), which is in agreement with previously reported results (28) and suggests a completely solvent-exposed tryptophan residue in the buffer conditions studied. Increasing doses of zymosan yielded a blue shift of the fluorescence, indicating peptide folding and consequent hiding of the aromatic residue. To determine the binding affinity between TCP-25 and zymosan, we reported the shift in emission maxima (λ_max_) as a function of increasing concentrations of zymosan (Fig. 4C). Using this methodology, the binding affinity (*K_d_*) found was determined to be 64.0 ± 10.6 μg/ml.

### Antifungal activity of TCP-25 *in vivo*.

**FIG 5:**
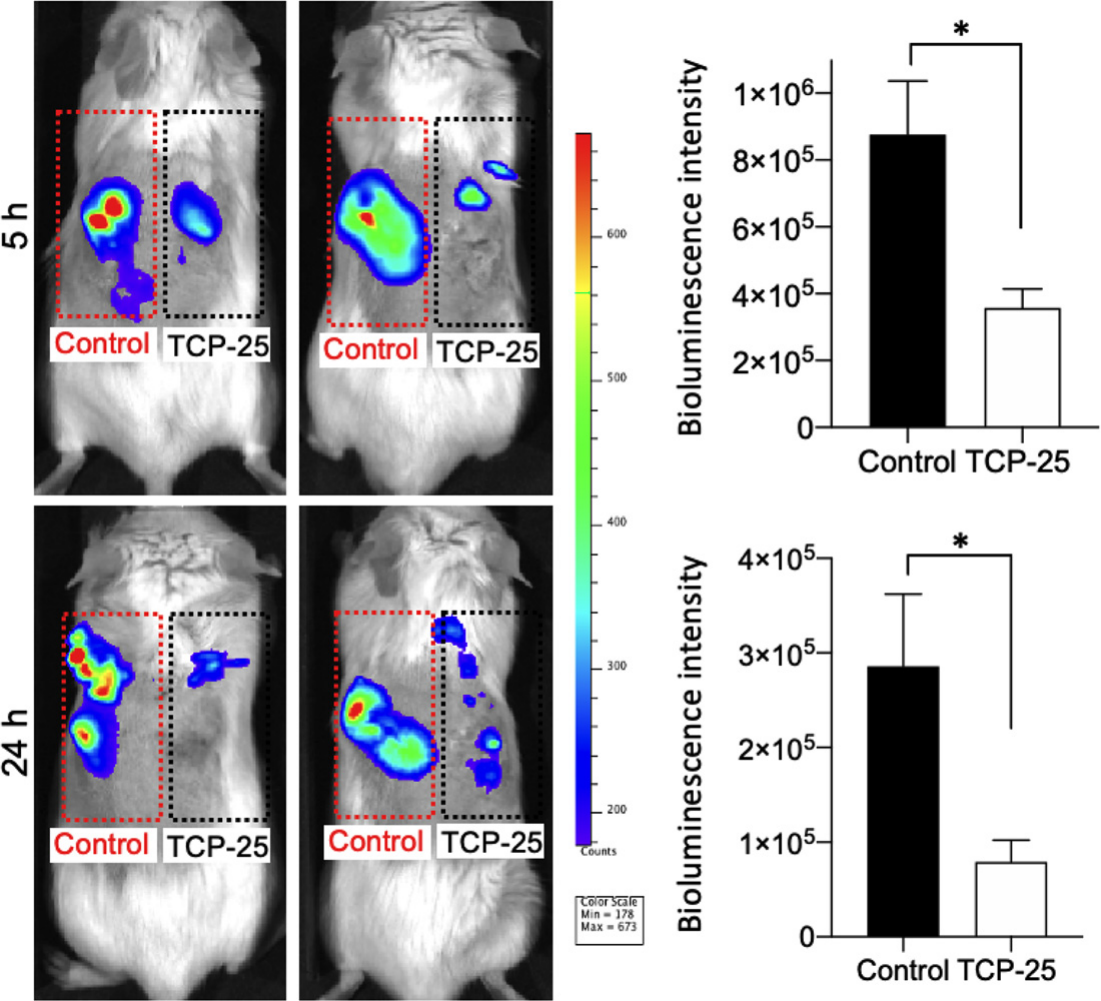
Fungicidal activity of TCP-25 *in vivo*. The effects of TCP-25 in a mouse model of subcutaneous microbial contamination are illustrated. Polyurethane (PU) discs with only buffer (left) or TCP-25 (right) were inserted subcutaneously in BALB/c mice dorsum. Immediately after, the discs were contaminated with bioluminescent *Candida* (5 × 10^6^ CFU). Bioluminescence intensity was analyzed using the IVIS bioimaging system. Representative images show yeast luminescence at 5 and 24 h postinfection. The bar chart shows measured bioluminescence intensity emitted by *Candida* at 5 and 24 h postinfection. All *in vivo* data are presented as the mean ± SEM (*n *=* *5 mice per group for 5 h and 4 mice per group for 24 h). *, *P ≤ *0.05; *P* values were determined using the Mann-Whitney U test.

The antifungal activity of the peptide *in vivo* was also studied using a mouse model of subcutaneous microbial contamination (Fig. 5), where a polyurethane implant material with or without TCP-25 was challenged with *Candida*. This model has previously been used for analyses of subcutaneous infections with Staphylococcus aureus and Pseudomonas aeruginosa (27, 42). For this, polyurethane (PU) discs with buffer or TCP-25 were implanted subcutaneously in BALB/c mice and immediately contaminated with bioluminescent *Candida* (5 × 10^6^ CFU). The yeast load was evaluated after 5 and 24 h by *in vivo* bioimaging. The results for both time points showed that the TCP-25 disc had significantly lower levels of *Candida* compared to those on the control PU disc, confirming that TCP-25 retains its anti-infective function against *Candida* in *in vivo* settings.

## DISCUSSION

*Candida* is an opportunistic pathogen, yielding infections that are resistant not only to our own immune defense systems but also to existing drugs (43). Similarly to bacteria, *Candida* can induce both infection and inflammation. Therefore, an effective treatment must address these two important aspects of the infection process. Many of the drugs available today focus on either antimicrobial or anti-inflammatory effects, and rarely are the two activities combined in the same drug. Our group has previously shown that TCP-25 has a dual action in the context of bacterial infections (24, 27, 28, 31). However, it remained unknown whether TCP-25 is also active against *Candida*. The main goal of this study was, therefore, to obtain insights into the antimicrobial and anti-inflammatory activity of TCP-25 against the yeast *Candida*.

Using several *in vitro* and *in vivo* models, we demonstrated that TCP-25 was able to target *Candida*. We did, however, observe a lower killing effect in VCA for the yeast forms compared to previous results against bacteria. Indeed, <5% survival was seen in Escherichia coli at 6 μM the peptide (24), instead of the ∼85% survival for C. albicans at the same dose of TCP-25 (Fig. 1). Therefore, to be effective against fungi, higher concentrations of TCP-25 were required. This could be explained by analyzing the cytoplasmic membrane of two pathogens. Indeed, the charge and sterol composition between E. coli and C. albicans are very different (16). Although the yeast membrane has a higher negative charge than mammalian cells, the bacterial membrane is even more negatively charged (16). Because cationic interactions are important for TCP-25’s antimicrobial action (28, 31), the net charge of the pathogen’s membranes could therefore explain the differences in killing effect. Moreover, such reasoning could also explain why TCP-25 has no effect on C. glabrata, as this *Candida* species is genetically more closely related to Saccharomyces cerevisiae than it is to the other *Candida* species (44, 45). In this regard, it was previously reported that both species showed a marked resistance to histatin-5, another antifungal peptide (46–48). This resistance was attributed in both cases to the lack of histatin-5 translocation into the cytoplasm of the yeasts (49). In this context, it was interesting that we observed a similar binding of TCP-25 in C. albicans as observed for histatin-5, which has been shown to be internalized into the vacuoles through receptor-mediated endocytosis at low concentrations and in cytoplasm at higher concentrations (49). Whether TCP-25 acts via similar mechanisms to those of histatin-5 clearly needs to be explored in further studies. Other explanations for the requirement of higher doses for antifungal effects in comparison to those required for bacterial killing could be related to microorganism size. Indeed, a *Candida* cell is around 5 to 10 times larger than bacterial cells, and therefore higher concentration of antimicrobial peptide may be needed per cell. Hypothetically, this could be the reason TCP-25 is less effective against C. krusei than against other species of *Candida*—the average size of a C. krusei cell is 25 μm in diameter, while a C. albicans cell, for instance, has a diameter of 3 to 8 μm (50).

By using live/dead staining and TEM, we observed that treatment with TCP-25 produced a dose-dependent increase in membrane permeability and cell death in *Candida*. Previous studies have shown that TCP-25 induces leakage of cell content and consequent death of bacteria (24). Live/dead staining, binding studies with TAMRA-labeled peptide, and TEM imaging studies showed that TCP-25 could potentially utilize the same mechanism of action in killing *Candida* as for bacteria, by binding the cellular membrane and then dose dependently increasing membrane permeability of the fungus, analogously to results with other membrane-active peptides (49). These data on TCP-25 are also in agreement with other studies (51). For example, a peptide from an archaeal protein (VLL-28) was able to bind to the cell wall of 10 clinical isolates of *Candida*; moreover, it was active toward the biofilm of some *Candida* species (52). The AMP from Musca domestica (MAF-1A) was shown to inhibit C. albicans growth via multitargets in the fungal cells (53). A 12-mer amino acid fragment of histatin-5, P-113, possessed strong candidacidal activity and showed no adverse effects in clinical trials (54–58).

*Candida* infection leads to activation of the immune system and subsequent inflammatory responses. This inflammation is important for infection clearance and the healing process, but overactivation of the inflammatory response might be deleterious if not controlled, further complicating the process of healing (59, 60). For example, biofilm production by *Candida* in wound healing promotes a chronic state of inflammation by creating a physical barrier and providing the wound with low oxygen and low pH, in turn delaying the wound-healing process (61). Candida can also lead to localized long-term inflammatory processes, such as vaginitis (62), stomatitis (63), and paronychia (64). Of particular relevance is that the yeast often colonizes and infects immunocompromised individuals. Thus, *Candida* infections are seen in cancer patients undergoing treatment with cytostatic drugs (65), in immunocompromised individuals with HIV (66), and in patients with severe burns (67). The consequences of uncontrolled infection and inflammation can be so severe that they cause organ failure and lead to death (68). Therefore, an effective treatment should suppress this excessive inflammation in addition to killing *Candida* itself. Similar to bacteria, during an infection, yeast releases the cell wall component zymosan (69). Several studies have shown that zymosan binds to TLR2 on the surface of immune cells and, thereby, induces the expression of proinflammatory cytokines (34, 70). TCP-25 blocks the proinflammatory effects of various bacterial ligands (24, 28, 31, 71). To also study the roles of the peptide in relation to yeast infections, we therefore studied TCP-25’s anti-inflammatory activity using THP-1 monocytes exposed to zymosan or HKCA, human blood exposed to zymosan, and mice stimulated with zymosan or HKCA. We found that TCP-25 was able to attenuate NF-κB signaling in THP-1 cells *in vitro*, as well as *in vivo* in NF-κB reporter mice. Moreover, the peptide reduced the subsequent cytokine release in blood stimulated with zymosan and in an experimental mouse model of zymosan-induced peritonitis. Considering the results with THP-1 cells, it is notable that a lower concentration of LPS (100 ng/ml) compared to that of zymosan (10 μg/ml) induced a higher NF-κB activation, indicating that the monocytes are more sensitive to LPS. In spite of that, we observed that TCP-25 was not reducing zymosan-NF-κB activation to the same extent as in the case of LPS. This observation could be due to the fact that zymosan is known to trigger inflammation through dectin-1, apart from TLR-2 (72).

Previous studies have shown that TCP-25 targets LPS and CD14, leading to the inhibition of inflammation during bacterial infection (28). In this study, we show that TCP-25 binds to zymosan, inducing a change in the secondary structure of the peptide, as indicated by CD spectra, and a shift in emission maxima in intrinsic fluorescence of TCP-25. When TCP-25 binds to LPS, it adopts an α-helical structure (28). The result obtained with zymosan, on the other hand, shows an evident change of the peptide structure, but the helix is not so well defined, although a concentration of zymosan 2 times higher than that of LPS was used. These data suggest a lower affinity of zymosan to TCP-25. Indeed, the *K_d_* for LPS and TCP-25 was found to be 20.0 ± 2.9 μg/ml (28), while that for zymosan and TCP-25 was 64.0 ± 10.6 μg/ml. This is in agreement with previous observations that higher concentrations of TCP-25 are needed to kill *Candida* than to kill bacteria.

*Candida* infection on implantable medical devices is associated with very high mortality rates (73–75). Unfortunately, it is only rarely cured on site and in most cases require surgical removal (74). We demonstrated that PU discs coated with TCP-25 show a lower propensity to be contaminated by *Candida* when implanted in mice; this finding proves that TCP-25 as a coating material could be used to prevent not only bacterial (42) but also yeast infection.

In summary, we here, for the first time, demonstrate a previously undisclosed dual antimicrobial and anti-inflammatory activity of TCP-25 against *Candida*. The peptide exerted fungicidal activity *in vitro* against different *Candida* species and was able to inhibit *Candida*-induced inflammation *in vitro* and *in vivo.* The mechanism of action by which the peptide acts on *Candida* was found to involve membrane binding, followed by perturbation and disruption. The anti-inflammatory activity was dependent on binding between TCP-25 and zymosan, as demonstrated by structural changes and the different intrinsic fluorescence of the peptide in the presence of zymosan.

## MATERIALS AND METHODS

### Ethics statement.

The use of human blood was approved by the Ethics Committee at Lund University, Lund, Sweden (DNR2015/801). Written informed consent was obtained from all donors. The animal experiments were conducted according to national guidelines (Swedish Animal Welfare Act SFS 1988:534) and approved by the Laboratory Animal Ethics Committee of Malmö/Lund, Sweden (Permit Numbers: M8871-19 and M5934-19). The animals were housed under standard conditions of light and temperature and had free access to standard laboratory chow and water.

### Peptide and TLR-agonists.

The thrombin-derived C-terminal peptide TCP-25 (GKYGFYTHVFRLKKWIQKVIDQFGE) was synthesized by AmbioPharm, Inc. (North Augusta, SC). Tetramethylrhodamine (TAMRA)-labeled TCP-25 was synthesized by Biopeptide (San Diego, CA). The label was added to the N terminus of the peptide. The purity of all peptides (over 95%) was confirmed by mass spectral analysis (Voyager matrix-assisted laser desorption ionization–time of flight mass spectrometer; Applied Biosystems, USA).

Lipopolysaccharide (LPS) from Escherichia coli (serotype 0111:B4) and laminarin from Laminaria digitata were purchased from Sigma-Aldrich (Saint Louis, MO). Zymosan from Saccharomyces cerevisiae was purchased from InvivoGen (San Diego, CA).

### Culture of yeast.

Candida albicans (900228), C. parapsilosis (90018), C. krusei (6258), and C. glabrata (90030) were purchased from the American Type Culture Collection (ATCC), *C. lipolytica* was kindly provided by Andreas Sonesson (Lund University), and the SKCA23-*ACTgLuc* strain was the gift of Patrick Van Dijck (VIB-KU Leuven). All yeast species were maintained by subculturing every 3 weeks on Sabouraud dextrose agar (SDA; Sigma-Aldrich, St. Louis, MO) plates. Before each experiment, one colony of yeast was inoculated in 5 ml of yeast extract-peptone-dextrose (YPD) medium (Sigma-Aldrich) and allowed to grow overnight at 29°C. The day after, the optical density at 620 nm (OD_620_) was measured using a spectrophotometer (Genesys 20; Thermo Scientific, Rochester, NY). When the culture had reached the mid-logarithmic phase, i.e., ∼0.6 OD, the medium was removed by centrifuging the culture at 5,600 × *g* (1-6P; Sigma, USA) for 10 min. The pellet was resuspended in 5 ml of 10 mM Tris at pH 7.4 and centrifuged once more. After washing, the pellet was resuspended in 10 mM Tris at pH 7.4 (0.6 OD/ml) to obtain a 1% solution of yeast, which corresponds to approximately 1 × 10^9^ CFU/ml.

### Heat-killed preparation of C. albicans.

C. albicans (900228) was grown as reported above, washed in 10 mM Tris at pH 7.4, and then resuspended in the same buffer at a final concentration of 5 × 10^9^ CFU/ml. For heat inactivation, the yeast suspension was incubated at 100°C for 1 h (39) or kept on ice for 10 min, then at 80°C for another 10 min, and then again on ice (38). Heat inactivation was validated by plating 50 μl of the treated inoculum on SDA plates after incubation for 24 h at 30°C (see Fig. S4 in the supplemental material).

### Viable count assay.

A 1% yeast solution of C. albicans and C. parapsilosis was prepared as reported above. Thereafter, the yeast suspension was diluted 1:5 in 10 mM Tris at pH 7.4. Different concentrations of TCP-25 (1 to 50 μM) were added to the yeast suspension (55 μl total volume) and incubated for 2 h at 37°C. Yeasts with only Tris buffer were used as a control. At the end of the incubation time, serial dilutions (1:10) of all samples were performed in a 96-well plate. Then, 10 μl of each dilution was plated onto SDA plates. The plates were incubated at 30°C for 48 h. The colonies from each dilution were counted, and the CFU/ml was estimated according to the following equation:

$$\text{CFU/ml}=\frac{\text{colony dilution factor}}{\text{vol.}\;\left(\text{per spot}\right)\;\text{on plate}}$$

### Radial diffusion assay.

As previously described, a 1% solution of different yeasts was prepared and further diluted (1:10,000) in 10 mM Tris at pH 7.4 to obtain a 1 × 10^5^ CFU/μl working suspension. A 10-μl aliquot of this solution was mixed with 5 ml underlay gel (10 g/liter agarose (Sigma-Aldrich), 300 mg/liter tryptic soy broth (Sigma-Aldrich), and 0.02% Tween (Sigma-Aldrich) in 10 mM Tris at pH 7.4) at 42°C. The gel was quickly poured onto a petri dish (100 mm × 15 mm) and, after solidification, wells of 4 mm in diameter were punched in patterns of 5 × 3. TCP-25 was dissolved in 10 mM Tris at pH 7.4 with concentrations of 10, 20, 50, and 100 μM. Aliquots (6 μl) of each sample were then pipetted into the wells. Tris buffer alone was used as a negative control. The petri dish was then incubated at 37°C for 3 h. After incubation, 5 ml of overlay gel (10 g/liter agarose and 60 g/liter tryptic soy broth) at 47°C was poured onto the plate. The plate was then incubated at 30°C for 24 h. After incubation, the diameter of the developed zones was measured, excluding the punch diameter (4 mm).

### Live/dead staining.

C. albicans and C. parapsilosis were prepared and treated as for VCA. After 2 h of incubation with the peptide, the yeasts were mixed 1:1 with a staining solution (Live/Dead BactLight kit L7007; Invitrogen, Eugene, OR). The staining solution was a combination of two dyes, 2.5 μl of component A (SYTO 9 green fluorescent nucleic acid stain) and 2.5 μl of component B (red fluorescent nucleic acid stain propidium iodide) dissolved in 1 ml of 10 mM Tris at pH 7.4. The samples were incubated for 15 min in the dark at room temperature (RT). The cells were spun down at maximum speed in a centrifuge (Eppendorf 5424) for 5 min, and then 80% of the supernatant was discarded, whereas the pellet was redissolved in the remaining volume. A 5-μl aliquot was transferred onto a slide and covered with an 18-mm square coverslip. Each sample was then analyzed with a fluorescence microscope (Axio Scope.A1with EC Plan‐Neofluar 20× and 40× objectives, AxioCam MRm camera, Zen 2.6 [blue edition] and acquisition software; Zeiss). Ten view fields (1 mm × 1 mm) per sample from three independent sample preparations were acquired.

### Uptake of TCP-25 by C. albicans.

C. albicans was prepared as for VCA and then treated with increasing doses of TAMRA-labeled TCP-25 (1 to 50 μM). After 2 h of incubation, the yeast was incubated with 5 μg/ml DAPI for 15 min in the dark at RT. The cells were spun down at maximum speed in a centrifuge (Eppendorf 5424) for 5 min, and then 80% of the supernatant was discarded, whereas the pellet was redissolved in the remaining volume. A 5-μl aliquot was transferred onto a slide and covered with an 18-mm square coverslip. Each sample was then analyzed with a fluorescence microscope (Axio Scope.A1, equipped with EC Plan‐Neofluar 100×/1.3 oil and using Zen 2.6 [blue edition] acquisition software; Zeiss). 10Ten view fields (1 mm × 1 mm) per sample from three independent sample preparations were acquired.

### Transmission electron microscopy.

C. albicans was prepared and treated as for VCA. After 2 h of incubation with the peptide, the yeasts were fixed on carbon-coated grids (400 copper mesh) for 30 s. The cells were then stained with 7 μl 2% uranyl acetate for 20 s and washed two times with 10 mM Tris at pH 7.4 for 30 s. The grids were rendered hydrophilic via glow discharge at low pressure in the air before being used as reported previously (30). We examined the mounted grids (pitch μM) by TEM (JEM 1230; Jeol, Japan) and 30 view fields (×4,200 magnification) from three independent sample preparations.

### Culture of THP-1 cells.

THP1-XBlue-CD14^+^ cells (InvivoGen) were cultured in RPMI 1640 medium, complemented with 10% heat-inactivated fetal bovine serum and 1% antibiotic-antimycotic (Invitrogen, Carlsbad, CA), 100 μg/ml of G418 (InvivoGen), and 200 μg/ml of Zeocin (InvivoGen) at 37°C with 5% CO_2_ supply and 95% humidity. Every third day, the monocytes were subcultured in fresh medium.

### NF-κB assay.

THP1-XBlue-CD14 cells were cultured as described above. Cells were centrifuged at 2,000 × *g* for 5 min, and then the supernatant was discarded. The pellet was resuspended in prewarmed growth medium without antibiotics (G418 and Zeocin) at 1 × 10^6^ cells/ml. Subsequently, 180 μl of cell suspension was seeded in a 96-well plate. The proinflammatory effect of zymosan was analyzed by treating THP1-XBlue-CD14 cells with increasing concentration of the yeast ligand (1 to 10 μg/ml). Then cells were incubated for 18 to 24 h at 37°C with 5% CO_2_ and 95% humidity. The day after, 20 μl of cell medium was transferred to a new plate, mixed with 180 μl Quanti-Blue reagent (InvivoGen), and further incubated for 1 h (the remaining cell suspension was used for the cell viability assay described below). The concentration of secreted SEAP, which is an indicator for NF-κB activation, was quantified by reading the absorbance at 600 nm using a spectrophotometer (Victor3; PerkinElmer). In another set of experiments, THP1-XBlue-CD14 cells were exposed to a fixed concentration of zymosan (10 μg/ml) with and without increasing concentrations of TCP-25 (1 to 10 μM). The cells were grown, and NF-κB activation was analyzed as reported above. In both experimental settings, cells treated with 100 ng/ml of LPS were used as a positive control and untreated cells as a negative control. To evaluate the proinflammatory effect of HKCA on THP1-XBlue-CD14 cells, the monocytes were incubated with 1 × 10^7^ CFU/ml of HKCA obtained as described above. Zymosan (10 μg/ml) was used as a positive control. NF-κB activation was analyzed as described previously. In the last set of experiments, THP1-XBlue-CD14 cells were treated with HKCA (performed at 80°C) in the absence or the presence of increasing doses of TCP-25 (1 to 10 μM), and then secreted SEAP was quantified as described above.

### Cell viability assay.

The THP-1 cells from the above-mentioned NF-κB assay were used to establish the viability of the monocytes after exposure to an increasing dose of zymosan or zymosan and TCP-25. In brief, 20 μl of MTT [3-(4,5-dimethylthiazol-2-yl)-2,5-diphenyltetrazolium bromide] solution (5 mg/ml) were added to the cells and incubated for 90 min at 37°C. MTT is a yellow tetrazolium salt that is converted to insoluble purple formazan crystals by metabolically active cells, which are used as an indicator. At the end of incubation, the supernatant in the wells was carefully removed, and the formazan-formed crystals were dissolved in 100 μl of dimethyl sulfoxide (Duchefa Biochemie, Haarlem, Netherlands). The plate was then incubated at RT for 30 min on a shaker at low speed to allow for the solubilization of all crystals. The absorbance at 550 nm was then measured. A positive control was obtained by adding, to the untreated cells, a lysis buffer (Thermo Scientific, Rockford, IL, USA) and incubating at 37°C for 40 min before adding MTT. The absorbance value for untreated cells was considered to be 100%, and values for other treatments are shown in comparison to the untreated cells.

### Blood assay.

Fresh venous blood from healthy donors was collected in tubes with 50 μg/ml lepirudin. The blood was diluted 1:4 with RPMI 1640-GlutaMax I (Gibco) without phenol red. A 1-ml aliquot of blood mixture was distributed in a 24-well plate. A fixed concentration of zymosan (5 μg/ml) was added to the wells in the presence or absence of different concentrations of TCP-25 (1 to 10 μM). The plate was incubated at 37°C for 24 h. After incubation, the content in the wells was transferred to tubes and spun down at low speed for 5 min. The plasma was collected and stored at −80°C before analysis.

### Cytokine assay.

The levels of TNF-α, IL-1β, and IL-6 in human plasma were measured after the blood experiment described above. The assay was performed by using a human inflammation DuoSet ELISA kit (R&D Systems, Minneapolis, MN) specific for each cytokine, according to the manufacturer’s instructions. Absorbance was measured at a wavelength of 450 nm. Data shown are mean values ± standard error of the mean (SEM) obtained from at least 4 independent experiments, all performed in duplicate. For mouse plasma and peritoneal lavage, IL-1β was assessed using the Mouse IL-1β DuoSet ELISA kit (R&D Systems), whereas TNF-α, IL-6, IL-10, MCP-1, and gamma interferon (IFN-γ) were assessed using a mouse inflammation kit (Becton, Dickinson AB) according to the manufacturer’s instructions.

### Zymosan-induced peritonitis model *in vivo*.

Zymosan-induced peritonitis in mice was induced as reported by Fujieda et al., with some modifications (35). Briefly, zymosan was resuspended in endotoxin-free water and autoclaved for 30 min. Male C57BL/6 mice (11 to 12 weeks, 22 ± 5 g; Janvier) were injected intraperitoneally (i.p.) with 1 mg zymosan per mouse. Thirty minutes after zymosan injection, 0.5 mg TCP-25 in 10 mM NaOAc (pH 5) or buffer alone (control) was injected i.p. into the mice. After 4 h of zymosan stimulation, the animals were deeply anesthetized by isoflurane, and the blood was collected by cardiac puncture. Subsequently, a peritoneal lavage (PL) was performed with 4 ml of ice-cold phosphate-buffered saline (PBS). Plasma and PL samples were stored at −80°C until analysis.

### Circular dichroism.

The change in the secondary structure of TCP-25, due to the addition of zymosan was investigated using circular dichroism (CD). Laminarin was used for a control. The measurements were performed on a J-810 spectropolarimeter (Jasco, Tokyo, Japan) equipped with a Jasco CDF-426S Peltier set to 25°C. Each measurement was repeated 5 times with a bandwidth of 1 nm. The spectra were recorded between 190 and 260 nm with a scan speed of 20 nm/min, using a quartz cuvette with a light path of 2 mm (Hellma GmbH & Co. KG, Müllheim, Germany). The measurements were carried out on samples containing 10 μM TCP-25 alone or in combination with 100 or 200 μg/ml of the respective glucans. The signal of 10 mM Tris at pH 7.4 with or without the addition of glucan at the respective concentration was subtracted from the spectra of each sample for normalization, and the signal was converted to mean residue ellipticity, θ (mdeg · cm^2^ · dmol^−1^) following the equation shown below:

$$\text{molar ellipticity}\;=\;\frac{\left(\theta_{222})(\text{MRW}\right)}{\left(10\times c\times l\right)}$$

where θ_222_ is the observed ellipticity at 222 nm in millidegrees, *c* is the concentration in g/liter, *l* is the path length of the cuvette in cm, and MRW is the mean residual weight, i.e., the molecular weight of the protein (in Da) divided by the number of amino acids.

α-Helical content was calculated according to a previously published equation (40), shown below:

$$\alpha -\text{helical content}=\frac{\left([\theta {]}_{222}\;+\;3,\;000\right)}{\left(-39,\;000\;+\;3,\;000\right)}$$

where 3,000 and −39,000 have been established previously as constants based on the helicity of poly-l-lysine, as described by Greenfield and Fasman (40).

### Intrinsic fluorescence.

To study the possible effects of zymosan on the tertiary structure of TCP-25, the intrinsic fluorescence of the peptide was evaluated. The peptide (10 μM) alone, or with increasing concentrations of zymosan (9.9 to 91.0 μg/ml), was excited at 280 nm, and the emission spectra were recorded between 300 and 450 nm using a Jasco J-810 spectropolarimeter equipped with an FMO-427S fluorescence module. Measurements were performed at 20°C, with a sensitivity of 885 V and bandwidth of 2 nm. A dark cuvette with a light path of 3 mm × 3 mm was used. Then, *K_d_* was calculated, reporting λ_max_ as a function of the concentration of the yeast ligand. The result is expressed as an average of 3 independent experiments ± SEM.

### Native polyacrylamide gel electrophoresis and Western blot.

TCP-25 (2 μg) was incubated with increasing doses of zymosan (0, 0.10, 0.25, 0.50, and 1.0 μg/ml) in 20 μl as the final volume for 30 min at 37°C. Then, 5 μl of loading buffer (4× loading buffer native gel; Life Technologies) was added, and the samples were loaded onto 4 to 16% bis-Tris native gels (Invitrogen, Carlsbad, CA). The run was performed at 150 V for 100 min. The peptides were subsequently transferred to a polyvinylidene difluoride (PVDF) membrane using a Trans-Blot Turbo system (Bio-Rad, Laboratories, Hercules, CA). TCP-25 was detected using polyclonal rabbit antibodies against the C-terminal thrombin epitope VFR17 (VFRLKKWIQKVIDQFGE, diluted 1:1,000; Innovagen AB, Lund, Sweden), followed by porcine anti-rabbit horseradish peroxidase (HRP)-conjugated antibodies (1:1,000; Dako, Glostrup, Denmark). The peptide was visualized by incubating the membrane with SuperSignal West Pico chemiluminescent substrate (Thermo Scientific, Rockford, IL) for 5 min, followed by detection using a ChemiDoc X-ray spectrometry (XRS) imager (Bio-Rad Laboratories).

### Mouse subcutaneous contamination model.

Ten- to twelve-week-old BALB/c male mice (Janvier) were used to study the effects of TCP-25 against C. albicans infection *in vivo*. To facilitate *in vivo* imaging with IVIS, C. albicans expressing the Gaussia princeps luciferase gene (strain SKCA23-*ACTgLuc*), which produces bioluminescence upon the addition of substrate coelenterazine (76, 77), was used. To prepare the inoculum, SKCA23-*ACTgLuc* was grown as for VCA, washed, and resuspended to obtain suspensions of 10^8^ CFU/ml. Mice were anesthetized using a mixture of 2% isoflurane (Baxter) and oxygen. Hair on the dorsum of the mouse was removed using a clipper. An approximately 5-mm cut was made on the skin of the mouse’s back on the left and right sides, and the tip of the scissors was used to create a small pocket. A 6-mm-diameter polyurethane (PU) disc (Mepilex transfer; Mölnlycke Health Care, Gothenburg, Sweden) was used for subcutaneous implantation. TCP-25 (500 μg in 25 μl sodium acetate buffer) or 25 μl of buffer alone was added to the PU disc. One disc with TCP-25 and another control disc were inserted in the left or right subcutaneous pocket. Using a pipette, each PU disc was then subcutaneously contaminated with 50 μl (5 × 10^6^ CFU) of SKCA23-*ACTgLuc* suspension. The skin wound was closed with a suture (VICRYLTM; Johnson & Johnson, Belgium), and the mice were immediately transferred to individually ventilated cages (IVC). The *in vivo* status of *Candida* infection was longitudinally monitored by measuring bioluminescence using an IVIS Spectrum imaging system (PerkinElmer). Prior to imaging, at various time points, 100 μl coelenterazine substrate (60 μM in Tris) was subcutaneously injected in the PU discs. Bioluminescence from the mouse was detected and quantified using Living Image 4.0 software (PerkinElmer).

### Mouse inflammation model.

Eight- to ten-week-old male BALB/c-Tg(NF-κB-RE-Luc)-Xen reporter mice (Taconic Biosciences, Albany, NY) were used to evaluate the anti-inflammatory effects of TCP-25. HKCA (100°C for 1 h) was used for subcutaneous injection (5 × 10^6^ CFU in 50 μl). Just prior to subcutaneous deposition, HKCA suspension was mixed with TCP-25 (500 μg in 50 μl of 10 mM NaOAc at pH 5.0). For controls, HKCA inoculum was mixed with 50 μl NaOAc buffer. Mice were anesthetized using a mixture of 2% isoflurane (Baxter) and oxygen. Hair on the dorsum of mice was removed using a clipper. HKCA alone or mixed with TCP-25 was then subcutaneously deposited on the left and right side, respectively, on the backs of transgenic BALB/c Tg(NF-κB -RE-luc)-Xen reporter mice. IVIS *in vivo* imaging was performed for the longitudinal evaluation of NF-κB activation. Mice were injected intraperitoneally with 100 μl of d-luciferin (150 mg/kg body wt; PerkinElmer) 15 min before the imaging. Bioluminescent signals were acquired and quantified using Living Image 4.0 software (PerkinElmer).

### Statistical analysis.

All experiments were performed at least 3 times. The results are presented as means ± standard deviation (SD) (for RDA, VCA, NF-κB activation, cell viability, CD, and intrinsic fluorescence) or SEM (cytokine assay and *in vivo* data). The data were analyzed using Prism (GraphPad Software, Inc., USA). *P* values were determined using one-way analysis of variance (ANOVA) with Dunnett’s multiple-comparison test if not otherwise specified. A *P* value of ≤0.05 was considered statistically significant.
