# Supplementary material for: Thrombin-Derived C-Terminal Peptide Reduces Candida-Induced Inflammation and Infection In Vitro and In Vivo
Source: Antimicrob Agents Chemother. 2021 Oct 18;65(11):e01032-21. doi: 10.1128/AAC.01032-21 (PMC8522777; doi:10.1128/AAC.01032-21)
Supplement: Supplemental file 1 — Supplemental figures. Download AAC.01032-21-s0001.pdf, PDF file, 8.1 MB [file aac.01032-21-s0001.pdf]

Supplementary Figures

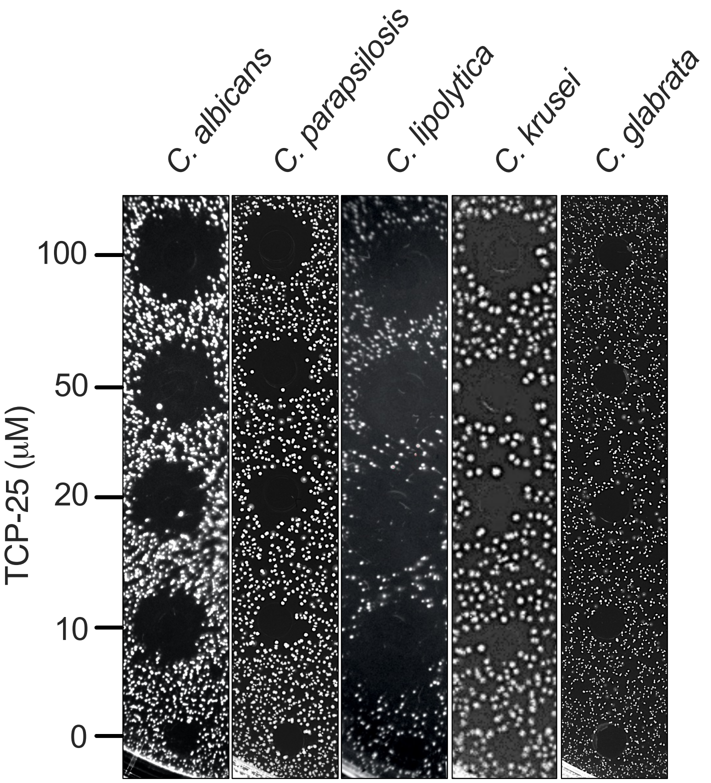

FIG S1 Antifungal activity of TCP-25 on different *Candida* species. Representative pictures of RDA illustrate the inhibitory effect of TCP-25 as zones of clearance on the species evaluated. TCP-25 was tested at 0, 10, 20, 50 and 100 μM.

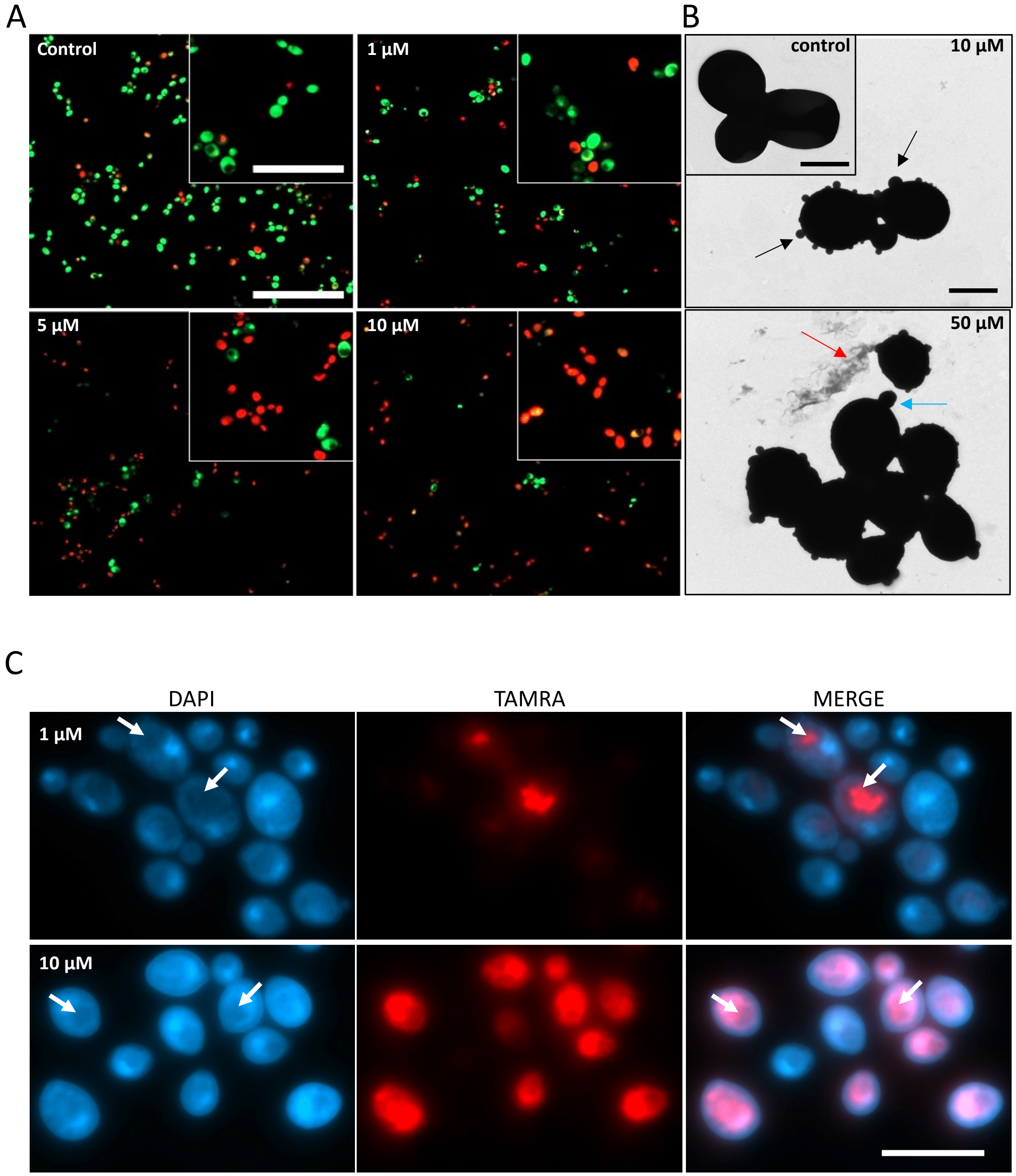

FIG S2 Permeabilization of *Candida* species by TCP-25. (A) Live/Dead assay was performed on *C. parapsilosis* treated with increasing concentrations of TCP-25 (0–10 µM). Representative picture for each treatment from three independent experiments is shown (n=3). In red dead yeast cells are visualized, in green alive cells. Scale bar is 50 µm for all images and 20 µm for the inserts. (B)

Representative TEM images are shown upon treatment of *C. albicans* cells with 2 different concentrations (10 and 50 μM) of TCP-25 (10 individual fields per experiment, n=3). In the insert, untreated yeast cells are shown. Black arrows indicate the blebs, the blue arrow the protrusions, and the red arrow the leakage of electron-dense material. The scale bar is 1 μm for all images. (C) *C. albicans* was treated with increasing concentrations of TAMRA-labeled TCP-25 (0-50 μM) for 2 hours. The images were taken after staining of yeast cells with DAPI. Representative zoomed pictures for 1 and 10 μM treatment (from Figure 2) are shown (n=3, at least 10 individual fields per experiment). Yeast cells are visualized in blue (DAPI); labeled TCP-25 is visualized in red (TAMRA). White arrows indicate the vacuole like structures. Scale bar is 10 μm.

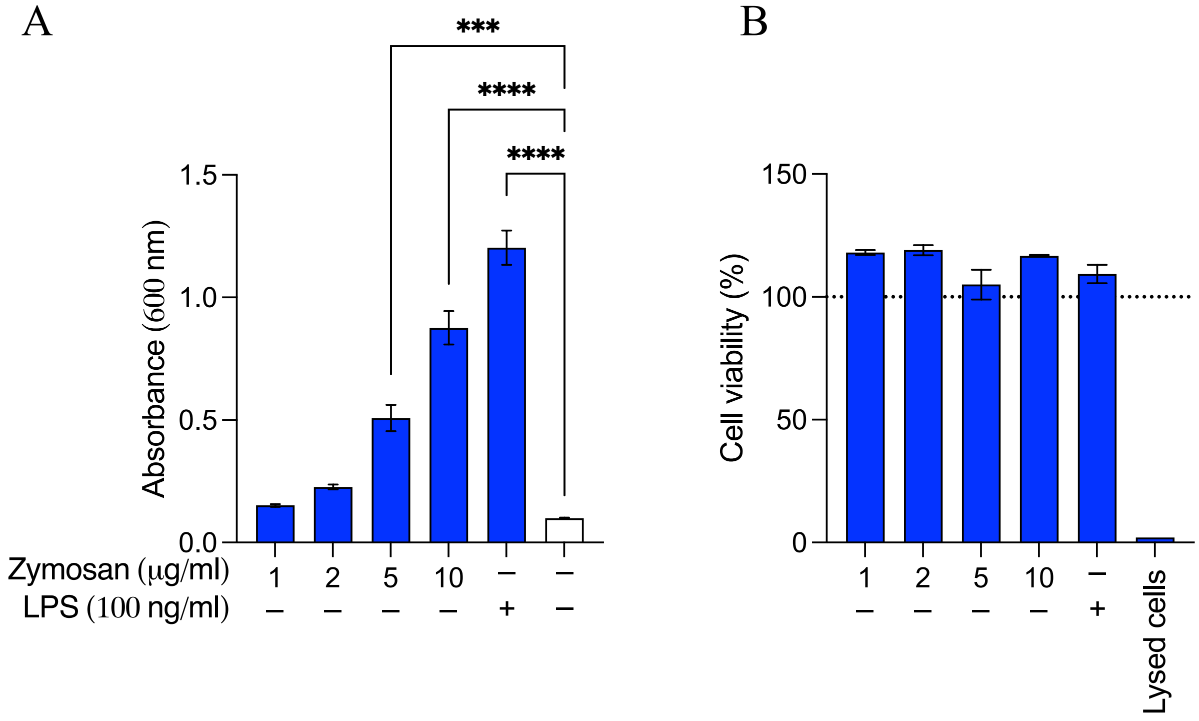

FIG S3 Pro-inflammatory effect of zymosan on THP1-XBlue-CD14 cells. Monocytes were incubated with increasing concentrations of zymosan and then NF-κB activation (A) and cell viability (B) were measured (n=3). LPS (100 ng/mL) was used as positive control. Lysed cells were used as negative control. The statistical significance is expressed in comparison to untreated cells and was obtained by using an ordinary one-way ANOVA followed by Dunnett's multiple comparisons test. ***$P \leq$ 0,001; **** $P \leq$ 0,0001. Dotted line in (B) indicates the vitality of untreated cells.

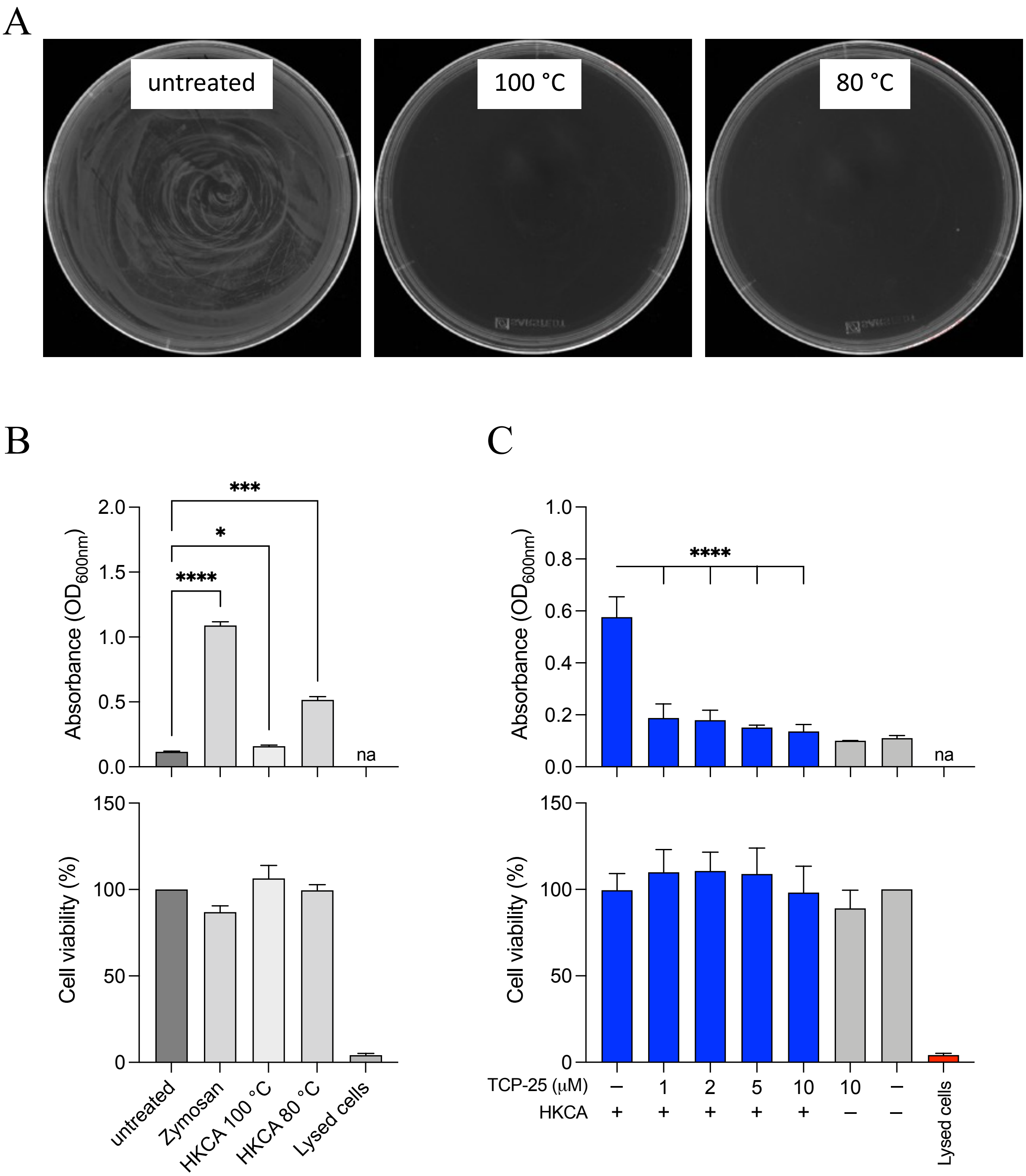

FIG S4 Pro-inflammatory effect of heat-killed preparations of *C. albicans* (HKCA) on THP1-XBlue-CD14 cells. (A) Representative images of SDA plates with untreated or HKCA generated at 100 or 80 °C, after incubation for 24 h at 30 °C. (A-B) THP1-XBlue-CD14 cells were incubated with: (A) 10 μg/ml zymosan or 10^7 cfu/ml of HKCA generated at 100 and 80 °C; (B) 10^7 cfu/ml of HKCA generated at 80 °C and increasing doses TCP-25 (1–10 μM). NF-κB activation and cell viability was

evaluated after incubation for 24 h at 37 °C. Lysed cells were used as negative control. Data are presented as mean ± SD (n=3). The significance is expressed in comparison to untreated cells (A) and cells treated with only HKCA (B) and was obtained by using an ordinary one-way ANOVA followed by Dunnett's multiple comparisons test. * $P \leq 0.05$, ***$P \leq 0.001$; **** $P \leq 0.0001$. na, not analysed.

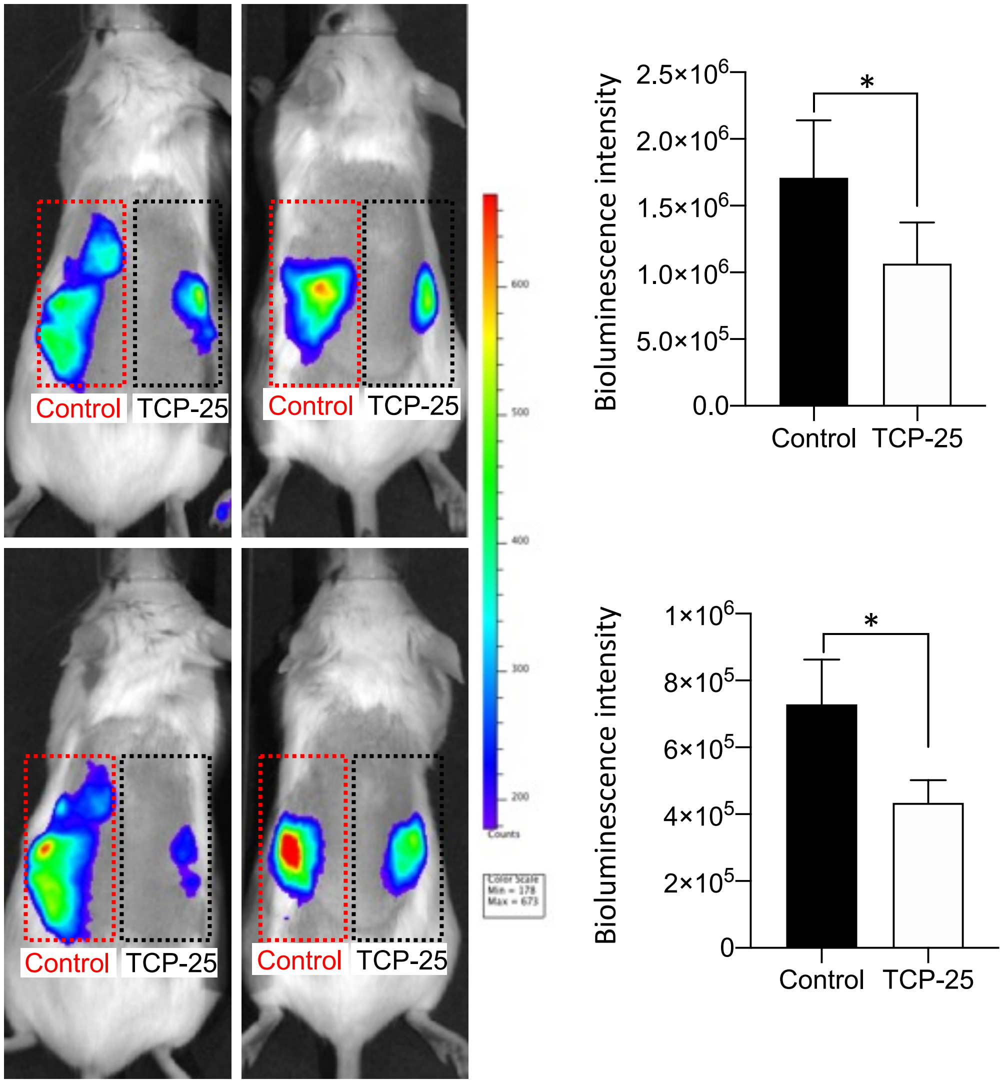

FIG S5 TCP-25 reduces HKCA-induced inflammation in mice. HKCA alone or mixed with TCP-25 was subcutaneously deposited on the left and right side, respectively, on the back of transgenic BALB/c Tg(NF-κB -RE-luc)-Xen reporter mice. IVIS imaging was used to monitor NF-κB reporter gene expression. Representative images show bioluminescence at 3 and 6 h after subcutaneous deposition. Bar chart shows the measured bioluminescence intensity. Data are presented as the mean ± SEM (n = 4). *P* values were determined using a t-test. * $P \leq 0.05$.

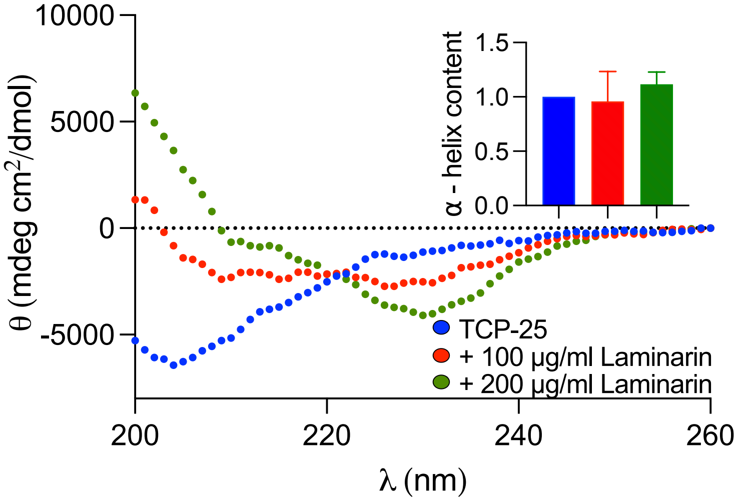

FIG S6 Structural changes of TCP-25 upon binding to laminarin. The secondary structure of TCP-25 (10 µM) before and after addition of 100 and 200 µg/ml laminarin was studied by CD. The measurements were performed immediately after the addition of laminarin. Representative spectra of 3 different experiments are reported (n=3). The α-helical content at 222 nm was calculated from the spectra obtained for each condition. The results are expressed as mean ± SD.
